# Supplementary material for: Patterns of Intron Gain and Loss in Fungi
Source: PLoS Biol. 2004 Nov 30;2(12):e422. doi: 10.1371/journal.pbio.0020422 (PMC532390; doi:10.1371/journal.pbio.0020422)
Supplement: Table S1 — Also available at http://genes.mit.edu/NielsenEtAl/. (4.3 MB ZIP). [file pbio.0020422.st001.zip › NielsenEtAl/html/411.html]

AN1989.1.NCU06761.1.MG07162.1.FG04413.1


```
 CLUSTAL W (1.82) Multiple Sequence Alignments - Introns Inserted


Sequence 1: NCU06761.1	576 aa
Sequence 2: MG07162.1	567 aa
Sequence 3: FG04413.1	572 aa
Sequence 4: AN1989.1	572 aa
Alignment Length: 590 aa
Number Identitical Residues: 293 aa
Alignment Score (without introns) 14441


MG07162.1 	------MPGTSRIPQSLRDGLISVKRNRPTSVVQLL--SIDL2LRNIVFTLFVLRWTRRA
NCU06761.1	------MPGTSRMPVSLRQSIDSVKRSRPRNPMQVLNLNVDL2IRNIVFFLFLLRWTRRA
FG04413.1 	MASPPHIGSLSRLPANLRGSIESVKRGGHNVSWPLL--NLDL2LRNVLLFLFFWRWSRRA
AN1989.1  	-------MASSVVPVALQNKLLGYGR-APSAQLAVL--NLDL2VRNIVFALFLFRYVRKT
          	        . * :*  *:  : .  *        :*  .:** :**::: **. *: *::

MG07162.1 	FWKLRGRGLLGWLAELYAAMRVTAYGWLLRAPGVRNKVRSEVDTALSKLSKKTLPP-PEA
NCU06761.1	LWKLKGRGLFGTLFELFTDARRILYGYFLRLPGVRTKVRAQIDDALTKMQAKMIPA-GQT
FG04413.1 	FWQLRGRGIIGSVVELYTNIRRILYGYFLRAPGVRGKVQQQVNESLVKLSDKLVPK-DQI
AN1989.1  	FYSLRGYGFFGSIHNVYLAIRLFLYSIFLRFPGVRGQVDKQVTAAIEGLESKLVANGPGV
          	::.*:* *::* : :::   *   *. :** **** :*  ::  ::  :. * :. .   

MG07162.1 	PRYLQIPKQGWTNEAVRAELESLANMDHTRWEDGYVSGAVYHGEDELISLQTDAYGKFTV
NCU06761.1	-RYLSLPKEGWTEEAVRKELEALATMDHTRWEDGYVSGAVYSGEDELLKLQTEAYGKFTV
FG04413.1 	-RYLTLPKEGFSHDVVRSELENLANMDHTRWEDGYVSGAVYHGEDELLALQTEAYGKFTV
AN1989.1  	TRYLTLPKEGWTHEQVRAELAKLGNMEHTRWEDGRVSGAVYHGGKDLLKIQAEAFEQFGV
          	.*** :**:*::.: ** **  *..*:******* ****** * .:*: :*::*: :* *

MG07162.1 	ANPIHPDVFPGVRKMEAEVVAM~VLSLFNAPVGAAGVSTSGGTESILMAVLSARQKAYNE
NCU06761.1	ANPIHPDVFPGVRKMEAEVVAM~VLSLFNAPPGAAGVSTSGGTESILMAILSARQKAYHE
FG04413.1 	ANPIHPDVFPGVRKMEAEVVSM~VLSMFHAPPGAAGTSTSGGTDSILMACLAARQKAYAE
AN1989.1  	ANPIHPDVFPGVRKMEAEVVAM0VLAMFHGPSDGAGVTTSGGTESILMACLAARNKARAE
          	********************:* **::*:.* ..**.:*****:***** *:**:**  *

MG07162.1 	RGVTEPEI2ILPETSHTAFRKAAEYYKIKVNFVACPAPDYQVDVSRVRRLINANTIMLVG
NCU06761.1	RGVTEPEM~IIPETAHTAFRKAAEYFNIKLHLVACPAPTYQVDTKRVARLINRNTIMLVG
FG04413.1 	RGVTEPEM2ILPETGHTAFRKAAEYFKIKLHLVACPAPEYQVDVNAVRRLINPNTVILVG
AN1989.1  	RGVTEPEM2IIPDTAHAAFIKASSYFGIKLHRVPCPAPDHKVDIAKVRRLINSNTVLLVG
          	*******: *:*:*.*:** **:.*: **:: *.**** ::**   * **** **::***

MG07162.1 	SAPNFPHGIIDDISGLSKLAVRYKIPLHVDCCLGSFLVPFLERAGFETRP-----FDFRL
NCU06761.1	SAPNFPHGIIDDISALSKLALRKKIPLHVDCCLGSFLVPFLDKAGFDSQP-----FDFRL
FG04413.1 	SAPNFPHGIIDDISALSKLALRKNIWLHVDCCLGSFLVPFLERAGFETLL-----FDFRL
AN1989.1  	SAPNFPHGIVDDIPALSRLATHYKIPLHVDCCLGSFVIALLKKAGFPSPYEEEGGFDFRQ
          	*********:***..**:** : :* **********::.:*.:*** :  .....**** 

MG07162.1 	KGVTSISVDTHKYGFAPKGNSTVLYRTAELRKYQYFVCPDWSGGVYASPGIAGSRPGALI
NCU06761.1	KGVTSISCDTHKYGFAPKGNSTVLYRTAELRSYQYFVDPSWSGGVYASPGIAGSRPGALI
FG04413.1 	KGVSSISCDTHKYGFAPKGNSTVLYRTAEHRKFQYFVSPDWSGGVYASPGMAGSRPGALI
AN1989.1  	PGVTSISVDTHKYGFAPKGNSVLLYRNKTYRSHQYFIYPDWSGGVYASPSVAGSRPGALI
          	 **:*** *************.:***.   *..***: *.*********.:*********

MG07162.1 	AGCWASMMAVGEAGYLAACVEIVGAAKKLVDHINASPSLAAELEIIGKPLSSVVAFRAR-
NCU06761.1	AACWASLMSVGEEGYLKSCTQIVGATKKLAEHIRSHPTLQQELEILGNPLVSVLAFQARG
FG04413.1 	AGCWASLMTMGESGYIDSCTKIVGTAKKIAEAIQTTPALGGELEIMGKPLVSVVAFTAR-
AN1989.1  	AGCWASLMSVGESGYIKSCLDIVNAAKKFESAINEDARLSPNLQVVGQPMVSVIAFESKN
          	*.****:*::** **: :* .**.::**: . *.  . *  :*:::*:*: **:** ::.

MG07162.1 	-NLNIYDIADAMGEKGWHLNSLQNPPAMHVAFTLPTAKVWERLATDLEACVEGEREKERA
NCU06761.1	-DLNIYDIADGMSSRGWHLNALQNPPAIHVAVTAPVAKNWERLAQDLEGVVEEEREKERV
FG04413.1 	-NLNVYDIADGMGTKGWHLNALQSPPAIHVAVTLPITKVWEKLITDLEAVVEEEREKERV
AN1989.1  	DAVDIYDIADDLSAKGWHLNALQSPPAMHVAFTIPTAAAVDTLISDLVAVVEKELEKAEE
          	. :::***** :. :*****:**.***:***.* * :   : *  ** . ** * ** . 

MG07162.1 	RIAE-------GKGAKGAAVGDSAALYGVAGSLPNKSVVVELANGFLDLLYKT
NCU06761.1	RQVEALKMGISGKTGKQKAAGDTAALYGVAGSLPNKSVVVDLARGFLDLLYKA
FG04413.1 	RLVE-------GKGTHGKAMGDASALYGVAGSLPNKSVVVDLASGFLDLLYKA
AN1989.1  	RKRQ-------GKSYVVK-RGDTSALYGVAGSMPDKSIVSRLAEGFLDTLYKA
          	*  :       **       **::********:*:**:*  ** **** ***:
```
